# Supplementary material for: Conditional chemoconnectomics (cCCTomics) as a strategy for efficient and conditional targeting of chemical transmission
Source: eLife. 2024 Apr 30;12:RP91927. doi: 10.7554/eLife.91927 (PMC11060718; doi:10.7554/eLife.91927)
Supplement: Figure 3—source data 3. [file elife-91927-fig3-data3.zip › Figure 3 source data3 part 1.zip!/Figure 3-Source data 3-part1/1175-9-CRISPResso_Report_rtnd62ko.zip!/CRISPResso_on_1175_11/1a.Read_barplot.pdf]

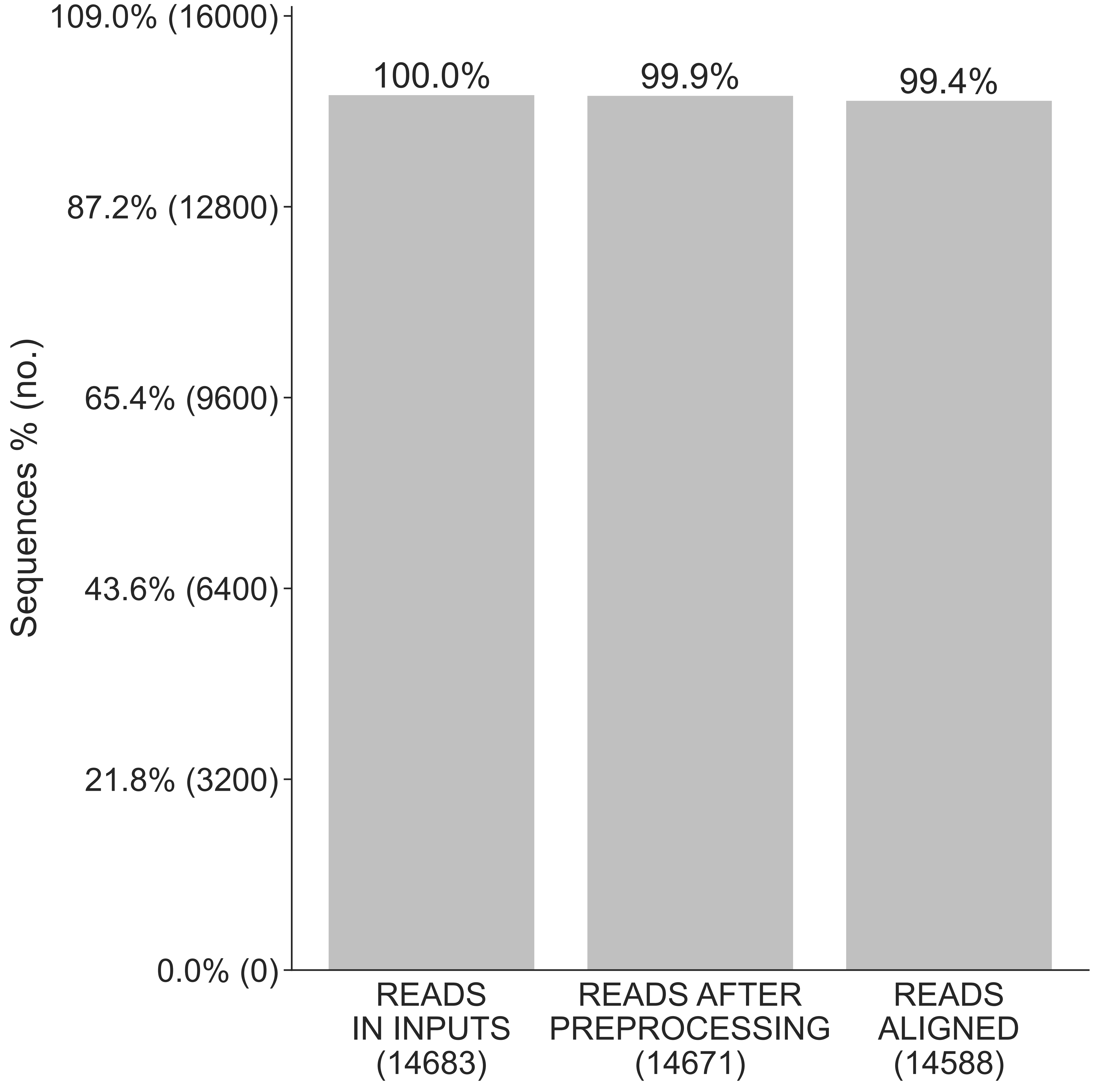

Sequences % (no.)
109.0% (16000)
87.2% (12800)
65.4% (9600)
43.6% (6400)
21.8% (3200)
0.0% (0)
100.0%
99.9%
99.4%
READS IN INPUTS (14683)
READS AFTER PREPROCESSING (14671)
READS ALIGNED (14588)
